# The immediate effects of iTBS on the muscle activation pattern under challenging balance conditions in the patients with chronic low back pain: A preliminary study

**DOI:** 10.3389/fnins.2023.1135689

**Published:** 2023-03-14

**Authors:** Jiajia Yang, Ruochen Fu, Zengming Hao, Nanhe Lin, Xue Cheng, Jinjin Ma, Yushu Zhang, Yan Li, Wai Leung Ambrose Lo, Qiuhua Yu, Chuhuai Wang

**Affiliations:** ^1^Department of Rehabilitation Medicine, The First Affiliated Hospital, Sun Yat-sen University, Guangzhou, China; ^2^Department of Urology, The First Affiliated Hospital, Sun Yat-sen University, Guangzhou, China; ^3^Guangdong Engineering and Technology Research Center for Rehabilitation Medicine and Translation, The First Affiliated Hospital, Sun Yat-sen University, Guangzhou, China

**Keywords:** chronic low back pain (CLBP), intermittent theta burst stimulation (iTBS), postural control, functional near-infrared spectroscopy (fNIRS), surface electromyography (sEMG)

## Abstract

**Background:**

The patients with chronic low back pain (CLBP) showed impaired postural control, especially in challenging postural task. The dorsolateral prefrontal cortex (DLPFC) is reported to involve in the complex balance task, which required considerable attentional control. The effect of intermittent theta burst stimulation (iTBS) over the DLPFC to the capacity of postural control of CLBP patients is still unknown.

**Methods:**

Participants diagnosed with CLBP received a single-session iTBS over the left DLPFC. All the participants completed the postural control tasks of single-leg (left/right) standing before and after iTBS. The activation changes of the DLPFC and M1 before and after iTBS were recorded by functional near-infrared spectroscopy (fNIRS). The activation pattern of the trunk [transversus abdominis (TrA), superficial lumbar multifidus (SLM)] and leg [tibialis anterior (TA), gastrocnemius medialis (GM)] muscles including root mean square (RMS) and co-contraction index (CCI) during single-leg standing were measured by surface electromyography (sEMG) before and after the intervention. The paired *t*-test was used to test the difference before and after iTBS. Pearson correlation analyses were performed to test the relationship between the oxyhemoglobin concentration and sEMG outcome variables (RMS and CCI).

**Results:**

Overall, 20 participants were recruited. In the right-leg standing condition, compared with before iTBS, the CCI of the right TrA/SLM was significantly decreased (*t* = −2.172, *p* = 0.043), and the RMS of the right GM was significantly increased (*t* = 4.024, *p* = 0.001) after iTBS. The activation of the left DLPFC (*t* = 2.783, *p* = 0.012) and left M1 (*t* = 2.752, *p* = 0.013) were significantly decreased and the relationship between the left DLPFC and M1 was significant after iTBS (*r* = 0.575, *p* = 0.014). Correlation analysis showed the hemoglobin concentration of M1 was negatively correlated with the RMS of the right GM (*r* = −0.659, *p* = 0.03) and positively correlated between CCI of the right TrA/SLM (*r* = 0.503, *p* = 0.047) after iTBS. There was no significant difference in the brain or muscle activation change in the left leg-standing condition between before and after iTBS.

**Conclusion:**

Intermittent theta burst stimulation over the left DLPFC seems to be able to improve the muscle activation pattern during postural control ability in challenging postural task, which would provide a new approach to the treatment of CLBP.

## 1. Introduction

Chronic low back pain (CLBP) is a common musculoskeletal disease (Cieza et al., 2021). In recent years, studies have suggested postural control ability in patients with CLBP was impaired (Berenshteyn et al., 2019). Changes in the morphology and activation of postural control muscles [i.e., transversus abdominis (TrA) and superficial lumbar multifidus (SLM)] are reported in the patients with CLBP (Hodges and Danneels, 2019). The co-contractions pattern of the agonist-antagonist muscle is a stiffening postural strategy, which could be estimated by calculating the co-contraction index (CCI) using surface electromyography (sEMG). The higher CCI is associated with poor postural control ability (Falk et al., 2022). A study found that patients with CLBP had a higher CCI and poor postural control (da Silva et al., 2019). Compared with healthy participants, patients with CLBP presented significantly poorer balance when executing single-leg standing tasks (da Silva et al., 2019). The single-leg standing represents a challenging part of postural control because, compared with bipedal stance, the area of the support is smaller and need more corrective movements to keep balance (Hertel et al., 2006). The impairment of postural control leads to spine instability, which contributes to the reoccurrence of low back pain (Brumagne et al., 2008a,b). Therefore, finding an approach to improve the capacity of postural control, especially in the challenging balance task like one-leg standing is very important for the patients with CLBP.

The ability of postural control is positively associated with the cortical activity. Previous studies showed that the primary motor cortex (M1), supplementary motor area (SMA), primary somatosensory cortex (S1), and dorsolateral prefrontal cortex (DLPFC) are involved in postural control (Teo et al., 2018; Solis-Escalante et al., 2019; Coelho et al., 2021; Nandakumar et al., 2021). Tsao et al. (2008) found that the activation onset of TrA during single rapid arm flexion was related to the location and the map volume of M1 in the CLBP participants. These findings indicate evidence to the reorganization of trunk muscle representation at the motor cortex (M1) in the patients with CLBP (Tsao et al., 2008). It was believed that the impairment of postural control contributes to the reoccurrence of low back pain (Brumagne et al., 2008a,b), one clinical study investigated the effect of rTMS over the M1 and DLPFC in patients with CLBP. Their results showed that rTMS over the left DLPFC resulted in pain relief, while rTMS over M1 could not effectively attenuate the pain (Freigang et al., 2021). Previous studies reported that the DLPFC is an important cortex in postural control. The connection of the DLPFC to M1 conveyed critical motor-related information for motor control (Duque et al., 2012; Wang et al., 2020). The DLPFC is a neural substrate involved in the planning of action-motor sequences, allocation of cognitive resources, and inhibitory control (Kaller et al., 2011; Teo et al., 2018; Coelho et al., 2021). Teo et al. (2018) reported that a significant positive association was observed between balance performance and increased the bilateral DLPFC activation particularly for more complex balance tasks. In the CLBP patients, the balance deficits present more obvious in the postural tasks with higher difficulty level (Berenshteyn et al., 2019). One study has showed that when the postural task became more difficult, more cognitive resources were required (Horak, 2006). This cognitive regulation was most likely related to the DLPFC (Ceko et al., 2015). A few studies had investigated the changes of the DLPFC in patient with CLBP. For instance, one study employing structural Magnetic Resonance Imaging (MRI) showed that there was a significant decrease in gray matter density and cortical thickness in the DLPFC in the patients with CLBP (Seminowicz et al., 2011; Ivo et al., 2013). Another functional MRI study reported aberrant functional connectivity related to intrinsic cognitive networks in the DLPFC (Ceko et al., 2015). These neuroimaging evidence suggested that improving postural control by regulating the activation of the DLPFC may be a potential approach to attenuate CLBP. Overall, the DLPFC plays an important role in postural control and be worth further researched.

Repetitive transcranial magnetic stimulation has been widely used in regulating brain activation and the plasticity of central nervous system thorough the stimulation of brain circuits (Klomjai et al., 2015). Studies have shown that rTMS can improve gait and balance in neurological diseases such as stroke, Parkinson’s, multiple sclerosis, and cerebral palsy (Dadashi et al., 2019; Ghosh, 2019; Tramontano et al., 2020). Intermittent theta burst stimulation (iTBS), which is a special mode of repetitive transcranial magnetic stimulation, has been demonstrated to regulate the excitability of the cerebral cortex and induce plastic changes in the cortex (Lowe et al., 2018). One study found that rTMS over the DLPFC could improve the postural control ability by in patients after stroke (Yu et al., 2022). After the intervention of iTBS on the left DLPFC, the performance during working memory tasks in healthy participants was improved by increasing the theta connectivity between the frontal and parietal regions (Hoy et al., 2016). However, there is a lack of research to explore the effect of iTBS over the DLPFC on the performance of challenging postural control of the patients with CLBP. Based on the previous findings that the activation of the left DLFPC was related to pain regulation process and postural control process (Lattari et al., 2017; Freigang et al., 2021) and iTBS over the left DLPFC could improve working memory task performance in compensating for vocal pitch perturbations (Hoy et al., 2016). Therefore, the left DLPFC was selected as the targeted iTBS stimulating side.

Functional Near-Infrared Spectroscopy (fNIRS) is a vascular-based functional neuroimaging technology that simultaneously measures the concentrations of oxyhemoglobin (HbO) and deoxyhemoglobin (HHb), which reflects the activation of the neural system (Leff et al., 2011). Compared with fMRI and electroencephalography, fNIRS is less sensitive to body movement, but with higher portability and more comfortability (Pinti et al., 2020). Therefore, fNIRS is more suitable to detect changes of neural activity during movement tasks (Pinti et al., 2020). In this study, we employed the fNIRS and sEMG to explore the immediate effect of iTBS over the left DLPFC on challenging postural control in the patients with CLBP and its neural mechanism. We hypothesized that a single session of iTBS over the DLPFC could promote the function of the DLPFC, subsequently decrease the CCI and improve the muscle activation pattern during postural control in CLBP.

## 2. Materials and methods

### 2.1. Participants

Twenty CLBP patients (8 males and 12 females) were recruited in this study through recruitment advertisements and hospital outpatient department in the First Affiliated Hospital, Sun Yat-sen University. All the recruited participants were right-handed. The basic characteristics were as follows: age: 28.95 ± 4.58 years, height: 1.65 ± 0.08 m; weight: 60.3 ± 9.02 kg, BMI: 21.90 ± 1.94 kg/m^2^, back pain duration: 3.65 ± 3.39 years, Visual Analog Scale (VAS): 6.4 ± 1.43. The inclusion criteria were as follows: (1) Clinically diagnosed with CLBP, intermittent or persistent pain below the 12th rib of the lower back and lasted for ≥12 weeks; (2) Age between 18 and 40 years old; (3) Visual analog scale (VAS) score ≥3 points; (4) Right-handed; (5) No neurological disease; (6) No low back pain-related drug treatment in the past 3 months. Exclusion criteria were as follows: (1) Spinal stenosis, spondylolisthesis, vertebral fracture, osteoporosis, tumor, tuberculosis, severe or progressive scoliosis, or low back pain due to rheumatic immune/inflammatory disease; (2) Dysmenorrhea, postpartum low back pain or pregnancy; (3) With a history of back surgery in the past 2 years, or back or shoulder injury in the past 1 year; (4) Severe cardiovascular and cerebrovascular diseases, high blood pressure disease; (5) Cancer or unexplained weight loss; (6) Mental illness; (7) Cognitive dysfunction, illiteracy or communication disorder; (8) Other contraindications to transcranial magnetic stimulation (metals implants in the body, previous seizures or use of anticonvulsants) or other conditions that would not cooperate with this study. This study was performed under the principles of the Declaration of Helsinki and was approved by the Ethics Committee of the First Affiliated Hospital of Sun Yat-sen University. All participants completed an informed consent form before the experiment.

### 2.2. iTBS protocol

Intermittent theta burst stimulation was performed using a NS5000 Magnetic Stimulator (YIRUIDE Medical Co., Wuhan, China). Each participant was given a single-session iTBS over the left DLPFC. The iTBS protocol began with a 2-s burst train (totally 30 pulses), which repeats every 10 s. Each burst train consisted of 10 triplet pulses with an inter-burst interval of 0.16 s, thus the triplets fire at a rate of 5 Hz. Overall, each participant received 600 stimuli during a single-session iTBS. The scalp location of the left DLPFC was the F3 electrode of the EEG cap which was designed according to the International 10–20 system (Herwig et al., 2003). The intensity was 80% resting motor threshold. Resting motor threshold was determined as the lowest stimulation intensity of the TMS pulses on the left M1. This stimulation intensity could produce motor evoked potentials (MEP) ≥50 uV in the right first dorsal interosseous muscle (FDI) in five out of ten consecutive stimulations (Grossheinrich et al., 2009).

### 2.3. sEMG recording and data processing

Surface electromyography data of the bilateral TrA, SLM, tibialis anterior (TA), and gastrocnemius medialis (GM) were recorded by using the wireless surface EMG device (Trigno, Delsys, Inc., USA) with a sampling rate of 2 KHz. The electrodes were placed over the muscle belly after the skin preparation was performed by shaving and cleaning with peeling cream (Nuprep R^©^, Weaver and Company, Aurora, CO, USA) and alcohol. The orientation of the electrodes was parallel with the muscle fibers. The electrode placement of the target muscles, which referred to the previous study (Blanc and Dimanico, 2010). The TrA is 2 cm medial and inferior to the anterior superior iliac spine. The SLM is 3 cm from the midpoint line from the spinous processes of the L4 to L5 vertebrae. The TA is located at 1/3 of the distance between the tip of the fibula and the medial malleolus. The GM is located at the first lateral quarter from the tip of the head of the fibula to the lateral malleolus.

The sEMG data were processed in MATLAB 2013a (MathWorks, Natick, MA, USA). The raw data were bandpass filtered between 20 and 450 Hz in four orders, zero-lag Butterworth filters, followed by full-wave rectification and linear enveloped. Then, each sEMG envelope is normalized according to the baseline amplitude (during double-leg standing). During the single-leg standing, there would be postural interference when raising and lowering the legs at the beginning and the end of the task, which makes the sEMG signal fluctuate violently. The middle 10 s is the relatively stable to analyze the sEMG signal. So, the first 10 s and the last 10 s of the single-leg standing tasks were removed. Only the sEMG signals across 10–20 s time series were extracted and used in the data analysis. The root mean square (RMS) of the bilateral TA, GM, TrA, and SLM was calculated to reflect the muscle activation. The CCI of the bilateral TA/GM and TrA/SLM was calculated and averaged across the three single-leg standing tasks to reflect the postural muscle pattern according to the following equation (Mohr et al., 2018):


$$C\,C\,I=m\,e\,a\,n\,\left[\frac{M\,i\,n\,E\,M\,G\,\left(t\right)}{M\,a\,x\,E\,M\,G\,\left(t\right)}*\left(M\,a\,x\,E\,M\,G\,\left(t\right)+M\,i\,n\,E\,M\,G\,\left(t\right)\right)\right]$$

The *MinEMG (t)* and *MaxEMG (t)* represents the normalized activity intensity of the less active muscle or more active muscle separately for a certain sample *t*. The CCI of the paired muscles of the TA/GM and the TrA/SLM were chosen. The CCI of the TA/GM was chosen because it reflects the ankle muscle recruitment and modulation strategies in postural control (Vitali et al., 2021). The CCI of the TrA/SLM was chosen because the co-activation of the TrA/SLM is important for lumbar segmental stabilization and control (Matthijs et al., 2014).

### 2.4. fNIRS recording and data processing

**FIGURE 1:**
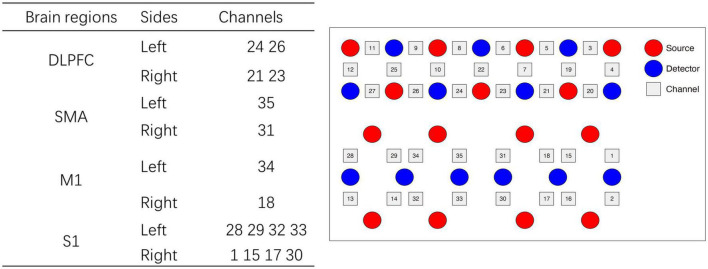
The configuration of the channel arrangement. DLPFC, dorsolateral prefrontal cortex; M1, primary motor cortex; SMA, supplementary motor area; S1, Primary somatosensory cortex.

During the single-leg standing tasks, the hemodynamic changes were monitored by a wearable fNIRS equipment (Nirsmart, Danyang Huichuang Medical Equipment Co., Ltd., China). The wavelengths were 730 and 850 nm and sampled at a frequency of 12 Hz. The fNIRS cap contains 24 source probes and 16 detector probes, which constitute 35 channels in accordance with the international 10–20 system. The selected regions of interest (ROIs) were the left and right DLPFC, SMA, M1, and S1 (Figure 1).

The original data collected were preprocessed by a MATLAB-based optical imaging software: NIRSPARK (Danyang Huichuang Medical Equipment Co., Ltd., China). The motion artifact and physiological noises in the raw optical density were filtered by 0.01–0.2 Hz bandpass (Hu et al., 2019). The filtered signals were converted into hemoglobin concentration based on the modified Beer-Lambert law. The hemoglobin concentration was extracted and the beta value of each ROI was calculated by general linear model (GLM) to reflect the hemodynamic response.

### 2.5. Experimental procedure

Each participant was required to complete single-leg standing tasks before and after iTBS. In our pilot study, we recruited 10 right-handed healthy participants (four participants underwent real iTBS stimulation, and another six participants underwent sham iTBS stimulation). The results showed that the sham iTBS over the left DLPFC did not change the HbO concentration in the DLPFC both during the left and right-leg standing tasks (*p* > 0.05). In addition, previous evidences from MEP and EEG have shown that there were no significant changes in the cortical excitability when applying the sham iTBS over the left DLPFC (Cao et al., 2018; Luo et al., 2023). Evidence from functional MRI also showed that a single-session iTBS over the left DLPFC did not change the brain activation and functional connectivity of DLPFC (De Wandel et al., 2020). In this study, we only tested the effects of real iTBS over the left DLPFC on patients with CLBP. In our study, all the recruited participants were right-handed. Previous studies have found that there was no difference in the balance performance and electromyographic measures between the dominant and non-dominant legs during single-leg stance (Lin et al., 2009; Kiyota and Fujiwara, 2014; Muehlbauer et al., 2014). The single-leg standing tasks were performed before and immediately after a single-session of iTBS. The order of standing on the left or right leg was random. The participants stood barefoot on the floor and stood with a single leg (left/right) for 30 s after a verbal cue. During the single-leg standing tasks, participants were instructed to keep their hands on their hips, but were not allowed to move or use their hands to keep balance. After the verbal cue “standing on left/right leg,” the non-supporting leg was asked to be raised to the knee level, with the toes pointing down and the calf paralleling to the ground, and the knee extension of the supporting leg is 0°. After that, they had a rest for 30 s. The participant repeated the single-leg standing task three times. The sEMG and fNIRS were carried out simultaneously during the balance tasks to assess muscle activation and brain activation.

### 2.6. Statistical analysis

Statistical analysis was conducted using SPSS version 25.0 (IBM SPSS Inc., Chicago, IL, USA) Windows software. The sEMG outcome variables including RMS and CCI, and the mean beta value and the HbO concentration were calculated across the three single-leg standing trials. Because of the small sample size in this study, we chose the Shapiro–Wilk test to test the normality (Razali and Yap, 2011). For the data with normally distribution, the paired *t*-test was used to test the difference before and after iTBS. Wilcoxon signed-rank test was used if the data was not normally distributed. The HbO concentration was averaged across the left and right ROIs. Pearson correlation analyses were performed to test the relationship between the HbO concentration and sEMG outcome variables (RMS and CCI). In the analysis of the beta value in the four ROIs (left and right DLPFC and M1) and the sEMG outcome variables (the RMS and CCI), we gave the FDR_BH_ correction using the MATLAB based on the Benjaminiand Hochberg’s method (Singh and Dan, 2006). *p* < 0.05 indicated a significant difference.

## 3. Results

### 3.1. sEMG results in single-leg standing tasks

**FIGURE 2:**
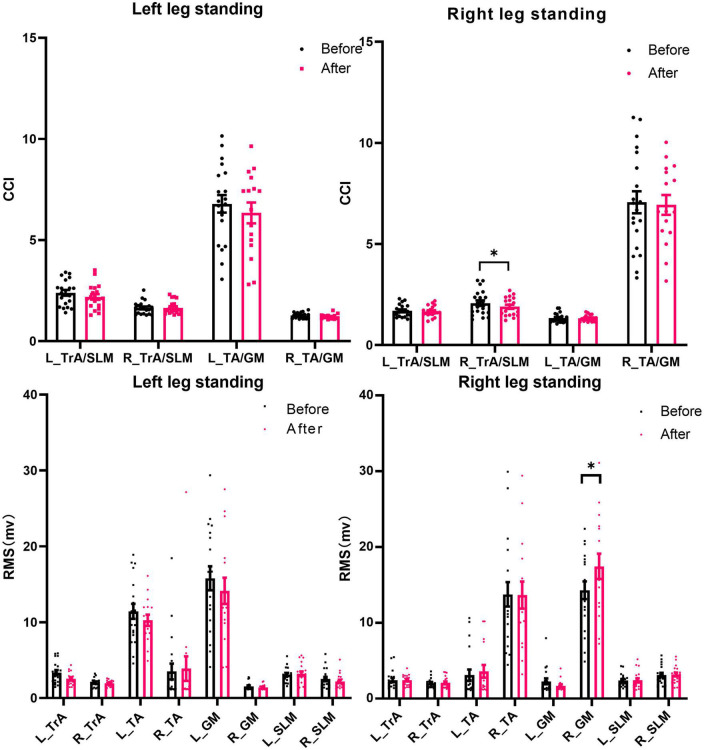
The CCI and RMS of postural muscles during single-leg standing tasks before and after iTBS over the DLPFC. RMS, root mean square; CCI, co-contraction index; TrA, transversus abdominis muscle; SLM, superficial lumbar multifidus; TA, tibialis anterior muscles; GM, gastrocnemius medialis muscles; DLPFC, dorsolateral prefrontal cortex; M1, primary motor cortex. *p < 0.05. Error bars indicate standard errors.

Compared with before stimulation, after iTBS over the left DLPFC, the CCI of the TrA/SLM and TA/GM was decreased. Although the decrease of the CCI in the right TrA/SLM showed significance (*t* = −2.172, *p* = 0.043). However, the CCI were not significant after the FDR adjustment (*p*_FDR_ = 0.086). The RMS of the right GM was significantly increased in the tasks of right leg standing (*t* = 4.024, *p* = 0.001, *p*_FDR_ = 0.008). There was no significant difference of the RMS or CCI before and after iTBS in the left-leg standing tasks (*p* > 0.05) (Figure 2).

### 3.2. Results of the brain activation before and after iTBS over the DLPFC in single-leg standing tasks

**FIGURE 3:**
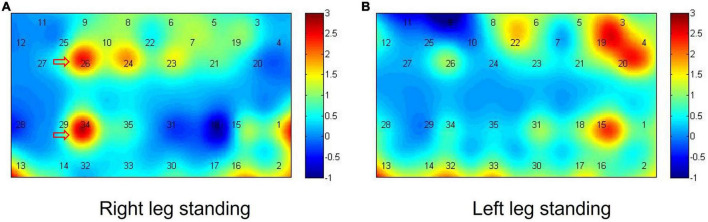
Activation maps in the single-leg standing tasks. **(A,B)** The difference of beta value before and after iTBS in the right-leg standing task **(A)** and the difference of beta value before and after iTBS in the left-leg standing task **(B)**.

In the right-leg standing tasks, after iTBS, the beta values changes over the left M1 (*t* = 2.752, *p* = 0.013) and the left DLPFC (*t* = 2.783, *p* = 0.012) were significantly decreased compared with before stimulation. In the left-leg standing tasks, all the ROIs showed no significant activation differences before and after iTBS (*p* > 0.05). The activation maps of the right and left-leg standing tasks were shown in the Figure 3.

**FIGURE 4:**
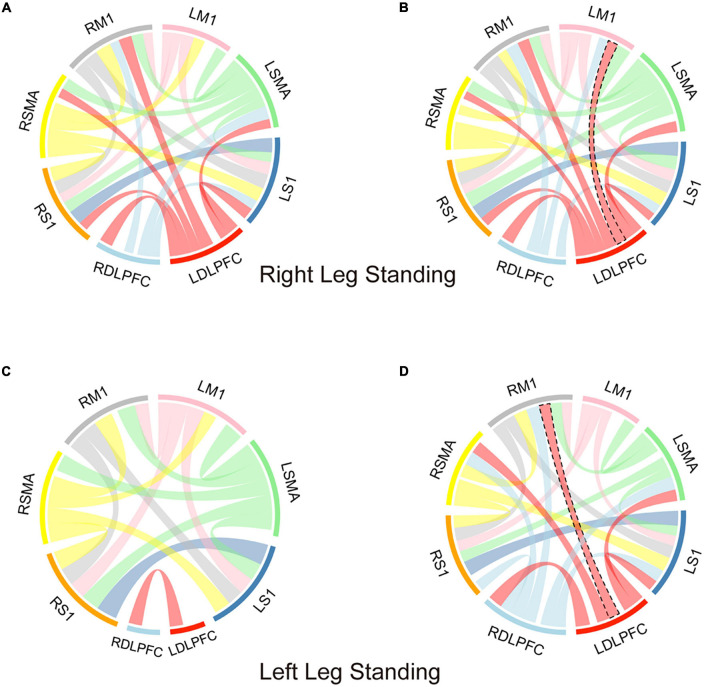
The correlation analysis of each ROIs in the single-leg standing tasks. **(A,B)** The correlation analysis of each ROIs before **(A)** and after **(B)** iTBS in the right-leg standing tasks. **(C,D)** The correlation analysis of each ROIs before **(C)** and after **(D)** iTBS in the left-leg standing tasks. DLPFC, dorsolateral prefrontal cortex; M1, primary motor cortex; SMA, supplementary motor area; S1, Primary somatosensory cortex. The connection band between the two ROIs indicates the correlation of the two ROIs was significant. The width of the joining band indicates the size of correlation coefficient. The black dotted line indicates the correlation difference before and after the iTBS.

In the right-leg standing tasks, before and after iTBS, the DLPFC showed significant correlation with the M1, SMA, and S1. Especially, compared with before iTBS, the left DLPFC showed significant correlation with the left M1 after iTBS (Before iTBS: *r* = 0.441, *p* = 0.052; After iTBS: *r* = 0.575, *p* = 0.014). In the left-leg standing tasks, before iTBS, the DLPFC showed no correlation with the M1, SMA, and S1 (*p* > 0.05). After iTBS, the DLPFC had significant correlation with the M1, SMA, and S1. Especially, the left DLPFC was significantly correlated with the right M1 (Before iTBS: *r* = 0.179, *p* = 0.707; After iTBS: *r* = 0.582, *p* = 0.012) (Figure 4).

The average HbO concentration of the left and right ROIs in the single-leg standing tasks was shown in the Table 1. Compared with before iTBS, the DLPFC (*t* = 3.712, *p* = 0.001) showed significant difference in the right-leg standing tasks after iTBS. There was no significant difference in all the ROIs in the left-leg standing tasks (*p* > 0.05).

**TABLE 1:** HbO changes (mmol/L) of four ROIs during single-leg standing tasks before and after iTBS over the DLPFC.

		Before (mean ± SD)	After (mean ± SD)	*t*-value	*p*-value
Right leg standing	M1	0.054 ± 0.026	0.044 ± 0.033	1.392	0.18
	DLPFC	0.077 ± 0.032	0.051 ± 0.035	3.712	0.001
	SMA	0.063 ± 0.039	0.050 ± 0.047	1.597	0.127
	S1	0.063 ± 0.043	0.061 ± 0.046	0.28	0.782
Left leg standing	M1	0.033 ± 0.043	0.040 ± 0.030	−0.674	0.509
	DLPFC	0.052 ± 0.046	0.032 ± 0.037	1.778	0.091
	SMA	0.036 ± 0.053	0.033 ± 0.044	0.183	0.857
	S1	0.045 ± 0.055	0.048 ± 0.039	−0.244	0.81

DLPFC, dorsolateral prefrontal cortex; M1, primary motor cortex; SMA, supplementary motor area; S1, Primary somatosensory cortex.

### 3.3. The correlation between the muscle activity and the brain region HbO concentration in the right-leg standing task

**FIGURE 5:**
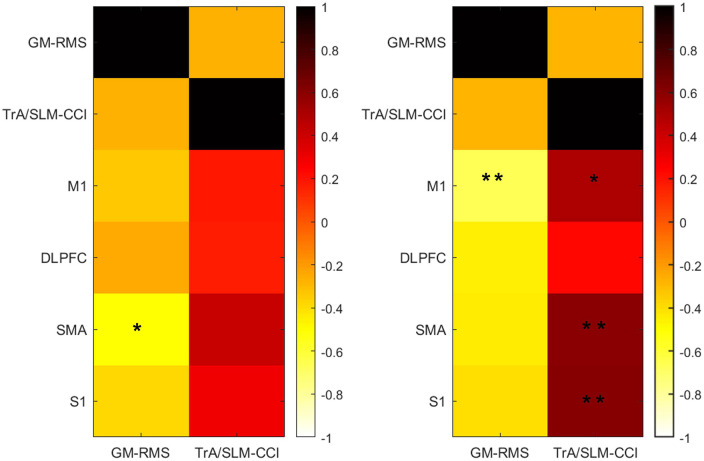
The correlation between the muscle activity and the HbO change among the ROIs before **(left)** and after **(right)** iTBS in the right-leg standing tasks. HbO, concentrations of oxyhemoglobin; RMS, root mean square; CCI, co-contraction index; TrA, transversus abdominis muscle; SLM, superficial lumbar multifidus; GM, gastrocnemius medialis muscles; DLPFC, dorsolateral prefrontal cortex; M1, primary motor cortex; SMA, supplementary motor area; S1, Primary somatosensory cortex. *p < 0.05, **p < 0.001.

Because there were significant changes both in the muscle activity and the brain region HbO concentration in the right-leg standing tasks. We gave a further correlation analysis. The results showed that the RMS of the right GM was negatively correlated with the HbO concentration of the M1 after iTBS (*r* = −0.659, *p* = 0.03). The CCI of the right TrA/SLM was positively correlated with the HbO concentration of M1 (*r* = 0.503, *p* = 0.047), SMA (*r* = 0.598, *p* = 0.017) and S1 (*r* = 0.603, *p* = 0.017) after iTBS (Figure 5).

## 4. Discussion

This is the first study from the brain and muscle activity level to investigate the effects of iTBS on the challenging postural control of CLBP patients. We found that iTBS over the left DLPFC could increase the postural control ability by increasing RMS and decreasing CCI. iTBS over the left DLPFC could decrease the activation of the left DLPFC and left M1, and increase the correlation between DLPFC and M1. The changes in the RMS and CCI were correlated with the brain activation of M1 after iTBS.

In the present study, we investigated muscle activation and co-contraction patterns in the lower limb and the deep trunk muscles during one-leg standing postural control task. We found the right lower limb muscle activity was increased and there was a decline in the co-contraction pattern of the trunk muscles during right-leg standing after iTBS over the DLPFC. These results suggested the muscle activation pattern during challenging postural control ability was improved. Impaired postural control exhibits a higher trunk muscle co-contraction (Kumai et al., 2022), which indicated postural control strategy alters to a co-contraction pattern to control body sway (Benjuya et al., 2004). In the CLBP individuals, a rigid back movement and a higher trunk muscle co-contraction pattern were adopted than those without CLBP for the fear of back pain. Co-contraction of trunk muscle subsequently leads to impaired hip flexion and a higher reliance on ankle strategy for maintaining balance (Arendt-Nielsen et al., 1996; da Silva et al., 2019; Koch and Hansel, 2019). The altered posture control strategy may be related to proprioceptive postural control. When CLBP patients adopted a body trunk stiffening strategy, they relied more on ankle muscle proprioception instead of paraspinal muscle proprioceptive control to maintain their posture (Brumagne et al., 2008b). Therefore, a single-session of iTBS over the DLPFC may be able to alter muscle proprioceptive strategies, and alter postural control.

In this study, the activity of the right GM was increased in right-leg standing after iTBS. This finding may be explained by the enhanced compensatory strategy related to challenging postural control. The compensatory strategy is the increased of the muscle activity around ankle joint for postural instability. In the patients with CLBP, the postural control ability is impaired (Benjuya et al., 2004), and the increased activity of ankle strategy-related muscles like GM suggests that this compensatory strategy exists (Jacobs et al., 2011).

This present study tested the single-leg standing performance and task-evoked neural activity simultaneously after iTBS. We found the activation of the DLPFC and M1 were decreased and the correlation was strengthened between the DLPFC and M1 after applying iTBS over the DLPFC in the patients with CLBP. Evidence from previous arterial spin labeling study showed an increased activation in the DLPFC and M1 in patients with CLBP (Wasan et al., 2011). Several recent structural and functional neuroimaging studies indicated that DLPFC is involved in the control of standing and balance by allocation of cognitive resources during the execution of motor control (Kaller et al., 2011; Teo et al., 2018; Coelho et al., 2021). Higher activation of the DLPFC represents that more cognitive resources were employed during the similar tasks (Sutoh et al., 2016). The attenuation of brain activity and enhancement of network connectivity together indicated a decrease in the need for cognitive control and more efficient neural code to control a motor task (Poldrack et al., 2005; Mazzoni, 2008; Wu et al., 2008). In the present study, the activation of the DLPFC were decreased, suggesting that iTBS over the DLPFC could make an efficient allocation of cognitive resources to achieve optimal motor control. Evidence from Choe et al.’s (2016) study also found that the decrease of HbO over the DLPFC exhibited a higher task performance since the increased efficiency of neural activity, which was in line with our finding. The DLPFC has various anatomical projections on the M1, and these two regions are thought to work closely together in motor tasks (Dum and Strick, 1991; Cao et al., 2022). We found the decreased DLPFC was correlated with decreased M1, suggesting there is an interaction between DLPFC and M1 after iTBS. This finding is similar with that reported in Wang et al.’s (2020) study, who used dual-coil paired-pulse transcranial magnetic stimulation to test the interactions between the ipsilateral DLPFC and M1. The results of Wang et al.’s (2020) study showed that there was a connectivity between DLPFC and M1, and the DLPFC dominantly contribute to inhibiting ipsilateral M1.

In our study, the co-contraction of agonist-antagonist muscles in the trunk was decreased, and the co-contraction was correlated with the HbO concentration of the M1, SMA and S1 after iTBS over the DLPFC in patients with CLBP. These findings suggested that the proprioceptive postural control may be related with the central neural system. Previous dynamic causal modeling studied the proprioceptive input for promoting motor activation and the result showed that the M1 or S1 could initiate feedback to SMA for proprioceptive motor integration (Nasrallah et al., 2019), which was in line with our findings. In our study, we also found the increased activity of GM was correlated with the decreased HbO concentration of M1. One study in rat model showed that the lesion of M1 influences the generation of compensatory behavior (Jones, 2017). The result is in line with our study that M1 is involved in the compensatory strategy, which more relies on the ankle strategy.

## 5. Limitation

There were some limitations in our study. Firstly, this is a preliminary study to investigate the immediate effect of iTBS over the DLFPC on challenging postural control. This study lacked the control group of sham iTBS, so the sEMG and fNIRS results were potentially influenced by learning effects or placebo effects. Future studies would consider the sham group in the study design and make our results more conclusive. Secondly, the sample size of this experiment was slightly small, which could reduce the robustness of the results. Thirdly, we only studied the immediate effect of iTBS over the left DLPFC without comparing the differences of iTBS effects between the left or right DLPFC. Future studies are needed to explore the effect of iTBS over the bilateral DLPFC on challenging postural control in the patients with CLBP. Fourthly, we did not evaluate the relationship between the changes in the muscle activation or brain excitability and clinical scale scores or cortical excitability like MEP. Because the previous preliminary study has found that no significant improvement in clinical scale related to pain after a single-session of iTBS (Lefaucheur et al., 2012). Exploring the treatment effects by applying a single or multi-session of iTBS for chronic low back pain and the relationship between the clinical scores and physiological parameters (EMG, HbO, or MEP) after a single or multi-session of iTBS is warranted in the future study. Lastly, the major objective of the present study was to compare the muscle activation pattern of each muscle before and after the iTBS intervention rather than the between-muscle differences. Therefore, we used the paired *t*-test to test the time effect on the muscle activation pattern. We used the FDR correction to control the potential probability of Type I error. Although we found there was a decrease tendency in the result of the CCI, it did not pass thorough the FDR correction. This may be due to the small sample size. Future studies should expand the sample size to make this result more conclusive.

## 6. Conclusion

Overall, iTBS over the left DLPFC seems to be able to enhance challenging postural control ability reflected by lower trunk muscle co-contraction and higher GM activity. The enhancement might be related to the deactivating DLPFC and M1 and strengthening the connectivity of DLPFC and M1 in the patients with CLBP. The present study reveals the mechanism of the DLPFC on the challenging postural control. Further investigation on the accumulative effect of iTBS over the left DLPFC is at least warranted.

## Data availability statement

The original contributions presented in this study are included in the article/supplementary material, further inquiries can be directed to the corresponding authors.

## Ethics statement

The studies involving human participants were reviewed and approved by the Ethics Committee of The First Affiliated Hospital of Sun Yat-sen University. The patients/participants provided their written informed consent to participate in this study.

## Author contributions

JY, RF, QY, and CW designed and conducted the study, data collection, and data analysis. WL and YL prepared the manuscript draft with important intellectual from JY and NL. ZH and NL provided statistical support in analyzing the data. JM, YZ, and XC had complete access to the study data. All authors approved the final manuscript.
